# Progressive exercise compared with best practice advice, with or without corticosteroid injection, for the treatment of rotator cuff disorders: statistical analysis plan for the Getting it Right: Addressing Shoulder Pain (GRASP) 2 × 2 factorial multicentre randomised controlled trial

**DOI:** 10.1186/s13063-020-04704-5

**Published:** 2020-09-07

**Authors:** Ioana R. Marian, Sally Hopewell, David J. Keene, Lucy Cureton, Sarah E. Lamb, Susan J. Dutton

**Affiliations:** 1 Oxford Clinical Trials Research Unit, Centre for Statistics in Medicine, Nuffield Department of Orthopaedics, Rheumatology, and Musculoskeletal Sciences, University of Oxford, Botnar Research Centre, Old Road, Oxford, OX3 7LD UK; 2 Kadoorie Research Centre, Nuffield Department of Orthopaedics, Rheumatology and Musculoskeletal Sciences and Oxford NIHR Biomedical Research Centre, Oxford University Hospitals NHS Foundation Trust, John Radcliffe Hospital, Oxford, UK; 3 Centre for Rehabilitation Research, Nuffield Department of Rheumatology, Orthopaedics and Musculoskeletal Sciences, University of Oxford, Oxford, UK; 4 College of Medicine and Health, Institute for Health Research, University of Exeter, St Luke’s Campus, Heavitree Road, Exeter, UK

**Keywords:** Statistical analysis plan, Randomised controlled trial, Factorial design, Rotator cuff, Corticosteroid injection, Exercise

## Abstract

**Background:**

The Getting it Right: Addressing Shoulder Pain (GRASP) trial assesses the clinical and cost-effectiveness of individually tailored, progressive exercise compared with best practice advice, with or without corticosteroid injection, in adults with a rotator cuff disorder. This article describes the statistical analysis plan for the GRASP randomised controlled trial.

**Methods/design:**

GRASP is a multicentre randomised controlled trial using a 2 × 2 factorial design. Adults aged ≥ 18 years with a new episode of shoulder pain related to a rotator cuff disorder, not currently receiving physiotherapy or being considered for surgery, are randomised (centralised computer-generated 1:1:1:1 allocation ratio) to one of four interventions: (1) progressive exercise (up to 6 physiotherapy sessions), (2) best practice advice (one physiotherapy session), (3) subacromial corticosteroid injection then progressive exercise and (4) subacromial corticosteroid injection then best practice advice. The primary outcome is the mean difference in Shoulder Pain and Disability Index (SPADI) total score over 12 months. Secondary outcomes are as follows: pain and function SPADI subdomains, health-related quality of life (EuroQol EQ-5D-5L), sleep disturbance, return to activity, global impression of change, health resource use, out-of-pocket expenses and work disability. Here, we describe in detail the following: sample size calculation, descriptive statistics of the primary and secondary outcomes, statistical models used for the analysis of the main outcomes, handling of missing data, planned sensitivity and subgroup analyses. This pre-specified statistical analysis plan was written and submitted without prior knowledge of the trial results.

**Discussion:**

Publication of the statistical analysis plan for the GRASP trial aims to reduce the risk of outcome reporting bias and increase transparency of the data analysis. Any deviations or changes to the current SAP will be described and justified in the final study report and any results publications.

**Trial registration:**

International Standard Randomised Controlled Trial Number ISRCTN16539266. Registered on 14 June 2016. EudraCT number 2016-002991-28. Registered on 12 June 2016.

## Background

Shoulder pain-related complaints are very common in the UK with an estimated prevalence of 14%, increasing with age and highest in those aged 60 or above [1, 2]. The most common attribution is the rotator cuff, accounting for 70% of cases, its disorders being associated with substantial disability and pain, affecting individuals’ work capacity and social activity. Evidence from small, short-term trials [3, 4] suggested that physiotherapist-prescribed exercise is effective for the treatment of rotator cuff disorders; however, there is a lack of evidence about the long-term clinical and cost-effectiveness [5]. Corticosteroid injections are commonly used to reduce pain and inflammation associated with rotator cuff disorders and are known to have a short-term benefit in the shoulder [6]; however, there are some concerns about their longer-term safety [7]. The combination of injection and physiotherapy has some evidence of an additive, but not interactive, short-term effect [6, 8, 9].

Getting it Right: Addressing Shoulder Pain (GRASP) is a factorial design randomised controlled trial assessing two interventions commonly used in the management of rotator cuff disorders in primary care: progressive exercise delivered by a physiotherapist and subacromial corticosteroid injection. Comparators are a best practice advice session with a physiotherapist and no injection. The study aims to assess which of these interventions, or combination of interventions, are most clinically and cost-effective.

This article reports details of the pre-specified statistical analysis plan as agreed by the Data Monitoring and Ethics Committee (DMEC) in September 2019 and has been prepared according to the published guidelines on the content of statistical analysis plans [10].

## Methods and design

### Trial design

GRASP is a 2 × 2 factorial, superiority, multicentre, randomised controlled trial. Eligible patients who consent to join GRASP are randomised to four intervention groups in a 1:1:1:1 ratio using variable block sizes of 4 and 8. The randomisation is stratified by centre, age group (18–35, > 35) and sex (male, female) via a secure, web-based system (Registration/Randomisation and Management of Product) at the Oxford Clinical Trials Research Unit and consistent with UK Clinical Research Collaboration-approved standard operating procedures. This system ensures prospective registration and allocation concealment until the point at which the patient enters the trial.

The four intervention groups are described as follows:

1. Progressive exercise programme (ProgEx): an individually tailored progressive home exercise programme prescribed and supervised by a physiotherapist involving up to six face-to-face sessions over 16 weeks
2. Best practice advice (BPA): one face-to-face session with a physiotherapist and a simpler home exercise programme supported by high quality self-management materials
3. Progressive exercise programme (as described above), preceded by a subacromial corticosteroid injection (ProgEx+I)
4. Best practice advice session (as described above), preceded by a subacromial corticosteroid injection (BPA+I)

Based on the assumption that there is no intervention interaction, the 2 × 2 factorial design allows two primary comparisons: (i) progressive exercise programme versus best practice advice and (ii) subacromial corticosteroid injection versus no injection, as listed in Table 1.

**Table 1:** GRASP intervention groups 2 × 2 factorial design

	No corticosteroid injection	Corticosteroid injection	***Effect of individually tailored progressive exercise programme***
**Individually tailored progressive exercise programme**	ProgEx	ProgEx+I	(*ProgEx*) + (*ProgEx+I*) vs. (*BPA*) + (*BPA+I*)
**Best practice advice**	BPA	BPA + I	
***Effect of corticosteroid injection***	(*ProgEx+BPA*) vs. (*ProgEx+I*) + (*BPA+I*)		

### Eligibility

Patients are eligible for this study if they are aged 18 years and above, have a new episode of shoulder pain (i.e., within the last 6 months) attributable to a rotator cuff disorder, not currently receiving physiotherapy, not being considered for surgery and able to understand spoken and written English [11].

### Blinding

The study participants and physiotherapists delivering the intervention cannot be blinded to the allocated treatment due to the nature of the intervention. The trial statistician and data entry personnel are also not blinded to allocation. The remaining members of the trial management team are blinded to allocation until the completion of data analysis. Full details of the trial design, study population and study procedures are available in the published GRASP protocol [11].

### Objectives

The primary objectives of the GRASP trial are to assess whether:

1. An individually tailored progressive home exercise programme prescribed and supervised by a physiotherapist provides greater improvement in shoulder pain and function over 12 months compared with a best practice advice session with a physiotherapist supported by high-quality self-management materials.
2. Subacromial corticosteroid injection provides greater improvement in shoulder pain and function over 12 months compared with no injection.

Secondary objectives are to investigate if there are any differences in the following: shoulder pain; shoulder function; health-related quality of life; psychological factors; sleep disturbance; return to desired activities including work, social life and sport activities; patient global impression of change; adherence to exercises; use of medication (prescribed and over the counter); time off work; health resource use (consultation with primary and secondary care) and additional out-of-pocket expenses. A within-trial health economic analysis will also be conducted.

### Outcomes

#### Primary outcome

The primary outcome for GRASP is *shoulder pain and function* measured over 12 months post-randomisation using the *Shoulder Pain and Disability Index* (*SPADI*), a numerical rating scale [12, 13]. The SPADI is a self-reported outcome consisting of 13 items divided in two dimensions measuring current shoulder pain (five-item subscale) and disability (eight-item subscale). Each item is scored on a 0–10 numerical rating scale, where 0 is the best and 10 is the worst score. The five-item pain subscale and eight-item disability subscales are totalled and standardised to a 0–100 scale, where a higher value denotes more pain and/or disability. Item-level imputation will be carried out for items where no more than 2 out of 5 items in the pain subscale are missing and no more than 3 out of 8 items in the function subscale are missing [13, 14]. A systematic review of outcome measurement sets for shoulder pain trials showed that SPADI is the most commonly used measure to assess pain and disability [15].

#### Secondary outcomes

The following secondary outcome measures in GRASP will be assessed at the time points described in Table 2.

**Table 2:** Time points at which outcomes are assessed

Outcome	Measurement	Time point
*Primary*		
Pain and function	Shoulder Pain and Disability Index (SPADI) 13-item total scale [16, 17]	0, 8 weeks, 6 months, 12 months
*Secondary*		
Pain	Shoulder Pain and Disability Index (SPADI) 5-item subscale [16, 17]	0, 8 weeks, 6 months, 12 months
Function	Shoulder Pain and Disability Index (SPADI) 8-item subscale [16, 17]	0, 8 weeks, 6 months, 12 months
Health-related quality life	EQ-5D-5L score [18]	0, 8 weeks, 6 months, 12 months
Psychological factors	Fear Avoidance Belief Questionnaire physical activity 5-item subscale [19] Pain Self-Efficacy Questionnaire (short form) insomnia [20]	0, 8 weeks, 6 months, 12 months
Sleep disturbance	Insomnia Severity Index [21]	0, 8 weeks., 6 months, 12 months
Global Impression of Treatment	Patient-rated Likert scale [22]	8 weeks, 6 months, 12 months
Return to desired activities	Patient-reported return to desired activities, including work, social life and sport activities	8 weeks, 6 months, 12 months
Exercise adherence	Patient-reported adherence to exercise	8 weeks, 6 months, 12 months
Work disability	Sick leave (days)	8 weeks, 6 months, 12 months
Healthcare use	NHS outpatient and community services (e.g., GP, additional physical therapy) NHS inpatient and day case (e.g., radiography, magnetic resonance imaging) Private health care services	8 weeks, 6 months, 12 months
Out-of-pocket expenses	Patient-related out-of-pocket expenses recording form	8 weeks, 6 months, 12 months

*SPADI pain* and *SPADI function* outcomes, as described above.

##### EuroQol EQ-5D-5L

The *EQ-5D-5L* is a validated, generic health-related quality of life measure consisting of 5 dimensions each with a 5-level answer possibility and a health thermometer scale [18]. The EQ-5D-5L can be used to report health-related quality of life in each of the five dimensions and each combination of answers can be converted into a health utility score where 1 represents perfect health and 0 indicates health states equal to death. The health thermometer visual analogue scale (EQ VAS) takes values between 0 and 100, where 0 represents worst imaginable health and 100 best imaginable health. It has good test-retest reliability and gives a single preference-based index value for health status that can be used for broader cost-effectiveness comparative purposes.

##### Fear Avoidance Belief Questionnaire Physical Activity (FABQ-PA)

FABQ is a validated measure of fear-avoidance behaviour [19]. FABQ-PA is a subscale of the FABQ, measuring fear-avoidance beliefs about physical activity using 5 items scored on a 0 (strongly disagree) to 6 (strongly agree) scale. The total score for FABQ-PA range is 0 to 24, with higher scores representing greater levels of fear-avoidance behaviour. Items 2, 3, 4 and 5 are summed for the final FABQ-PA score value.

##### Pain Self-Efficacy Questionnaire-2 (PSEQ-2)

The Pain Self-Efficacy Questionnaire (PSEQ) is a well-established 10-item measure of pain self-efficacy, a belief in one’s ability to carry out activities despite the pain [20]. PSEQ-2 is a 2-item short measure of pain self-efficacy. The 2 items reflect the confidence in one’s ability to work and lead a normal life despite the pain, on a 0 (not at all confident) to 6 (completely confident) scoring scale. The total PSEQ-2 score is summed from the 2 items giving a range between 0 and 12, with higher values representative of higher confidence levels despite the pain.

##### Insomnia Severity Index (ISI)

The ISI is a brief self-report measure of the participant’s perception of his or her insomnia, targeting the subjective symptoms, consequences of insomnia, as well as the degree of concerns or distress caused by those difficulties [21]. The ISI has seven items rated on a 0 to 4 scale and a total score is a summation of these items with a value ranging from 0 to 28. Higher score values are suggestive of a more severe insomnia. A detailed interpretation of the ISI total score is described as:

- 0–7 = No clinically significant insomnia
- 8–14 = Subthreshold insomnia
- 15–21 = Clinical insomnia (moderate severity)
- 22–28 = Clinical insomnia (severe)

*Global Impression of Treatment* (*GIT*) is a simple method of measuring change in health status with respect to the shoulder problems by charting self-assessed clinical progress on an 11-point scale answer ranging from − 5 (very much worse) to 5 (completely recovered), minimum detectable change of 0.45 points and MCID of 2 points [22].

*Return to desired activities* (*RDA*) is a self-reported outcome aiming to measure physical function during social life, recreational activities and work. RDA is an adapted version of the QuickDASH [23], using three questions with a 5-point Likert scale answer option, with lower scores indicating better function.

The participant’s *shoulder condition* is evaluated over three questions collecting information on rupture of the tendon in the shoulder, surgery and hospitalisation due to the rotator cuff disorder.

Other secondary outcomes including medication usage, work disability, healthcare use and out-of-pocket expenses will be analysed separately as part of a health economics analysis (further details in “Supplementary analyses and outcomes” section).

### Sample size

The target sample size is 704 randomised participants (176 per group). This assumes a baseline standard deviation of 24.3 [14] and is based on 90% power and 1% two-sided statistical significance to detect a minimally clinically important difference (MCID) of 8 points on the Shoulder Pain And Disability Index (SPADI) total scale [12]. This MCID is the equivalent to a standardised effect size of 0.33 requiring a sample size of 550 participants (Power Analysis and Sample Size 13, www.ncss.com). A potential loss to follow-up at 12 months of 20% allowance inflated the sample size to 688. The sample size was further inflated to take into account the potential for a small clustering by physiotherapist effect in the progressive exercise group. This used an interclass correlation (ICC) of 0.001, based on experience with individually tailored physiotherapy interventions [24], and the expectation that each physiotherapist would treat approximately 20 participants in the progressive exercise group. This led to an inflation of *f* = 1 + (*m* − 1) × ICC = 1 + (20 − 1) × 0.001 = 1.019 and increased the sample size to a total of 704 participants.

This sample size assumes no interaction effect between treatment groups and is powered for the two main effect comparisons:( i) progressive exercise versus best practice advice and (ii) corticosteroid injection versus no injection, when no interaction is present. However, if such an interaction effect does exist, this number of participants also provides 80% power and 5% two-sided significance to detect an interaction standardised effect size of 0.35 or more. The interaction effect will be tested before the main effect comparisons are undertaken. A non-significant interaction effect does not preclude a smaller interaction that this study is not powered to detect. 90% power and 1% two-sided significance was chosen to provide more convincing evidence of any treatment effects discovered. The DMEC undertook a blinded review of the sample size assumptions, particular to review the variability of the outcome measure, after 338 participants had been recruited and recommended a continuation of recruitment in line with the originally planned sample size.

### Statistical analysis

#### General analysis principles

A blinded analysis of data (not separated by intervention group) will be undertaken prior to the final data lock in order to assess the distribution of variables, missing data distributions and outliers. The intervention code will be added to the database after the data cleaning has been completed. The primary analysis population will be intention-to-treat (ITT) and will include all randomised participants in their allocated groups. Participant baseline characteristics will be reported by treatment group and overall. Descriptive statistics will be presented as numbers with percentages for categorical variables, and means and standard deviations or medians with interquartile range (IQR) for continuous variables, according to the data distribution. Statistical significance test or confidence intervals (CIs) will not be presented for differences between randomised groups in any baseline variable. A repeated measures linear mixed effects regression model will be used to estimate the overall treatment effects and the effects at each data collection time [25].

#### Statistical significance and multiple testing

The significance level used in the primary analysis is 1% and the corresponding 99% confidence interval (CI) will be displayed. We have dealt with multiplicity in an accepted method by having a single pre-specified primary outcome and pre-specified analysis plan. There will be no adjustment for multiplicity in the analysis. This factorial design trial answers two distinct research questions, and there is support in the literature that no adjustment is needed in such scenarios [26]. All secondary analyses will be considered as supporting the primary analysis; significance levels used will be 5% and 95% CI will be reported. It was intended that interim comparative analyses of the primary and secondary outcomes would not be carried out unless requested by the DMEC and none were requested. Any analyses not pre-specified will be exploratory in nature.

#### Description of study patient throughput

**Fig. 1:**
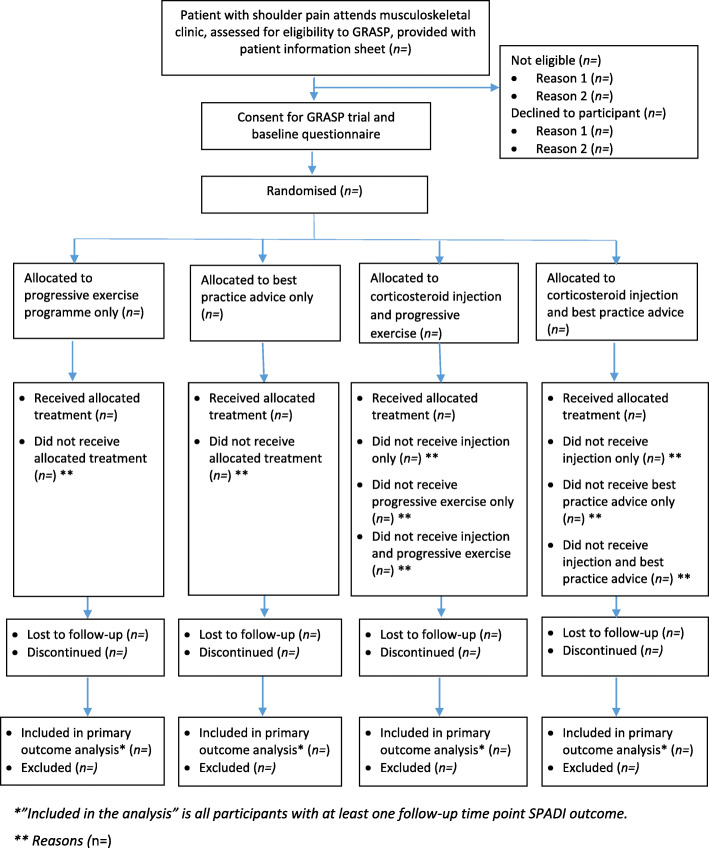
Study flow diagram template for GRASP trial (based on the CONSORT flow diagram)

The flow of participants through each stage of the trial, including numbers of participants approached; numbers eligible; numbers and reasons for ineligibility; numbers giving their consent and randomised, receiving intended intervention; completing the study protocol, and analysed for the primary outcome will be provided as per Fig. 1*.* Any participants who are randomised and retrospectively remove their consent for use of data will be listed in the participant flow diagram but their data will not be used in any further analyses.

#### Withdrawal from treatment and/or follow-up

The number (with percentage) of withdrawals from the trial and the numbers lost to follow-up for the primary outcome together with the associated reasons will be reported. Associations between loss to follow-up, baseline characteristics (sex; age; ethnicity; marital status; body mass index; smoking; shoulder affected; symptoms duration; hand dominance; work status; education; income; and Table 2 baseline outcomes) and intervention allocation will be explored. Any deaths (and their causes) will be reported.

#### Missing data

Throughout the trial, missing data are minimised by careful design, data management and training. Missing items within scales will be dealt with based on published instrument recommendations where these are available and no more than 10% of cases have missing data [27]. Missing data will be reported as number and percentage of participants missing all outcomes (e.g. loss to follow-up or withdrawal) or specific items only for each intervention group, together with reasons for missingness where known. Missing continuous primary and secondary outcomes will be handled as part of the likelihood-based estimation of the linear mixed model in the primary analysis assuming the missing at random (MAR) assumption holds [25, 28]. The robustness to departures from the MAR assumption will be assessed. Deviations from the MAR assumptions will be explored as part of the sensitivity analyses (described in the “Sensitivity analyses” section).

#### Description of compliance with intervention

A summary of the intervention allocated and received in each of the four groups (ProgEx, BPA, ProgEx+I, BPA+I) will be provided. This will include the number of sessions received and whether injections were given, where this is expected.

Compliance with the different aspects of the intervention will be reported by intervention group and summarised where possible with reasons for not receiving the assigned treatment. In the injection group, participants will be deemed compliant if they receive at least one corticosteroid injection. Participants randomised to the progressive exercise programme are intended to receive up to six sessions with a physiotherapist over 16 weeks. Not all participants will receive the full six sessions with a physiotherapist because the intervention allows for less sessions if the participant achieves their rehabilitation goals and are self-managing their shoulder problem. Compliance in terms of the progressive exercise intervention will be defined as the participant having been signed off as completing treatment by the physiotherapist (recorded on their treatment log) or having completed the maximum number of six sessions. Where participants are randomised to a treatment combination (ProgEx +I or BPA+I), compliance will take into account the described conditions for both treatments.

Deviations from intended treatment (non-adherence to the protocol) will be summarised for the randomised groups and will include incorrect treatment given or delayed treatment. The role of compliance on the treatment effect will be analysed using a Complier Average Causal Effect (CACE) approach (described in the “Supporting analysis based on compliance” section).

#### Analysis methods

The main analysis will be conducted as for two separate comparisons: (i) participants who receive progressive exercise compared to those who receive best practice advice, to determine the effectiveness of progressive exercise and (ii) participants who receive subacromial corticosteroid injection compared to those who do not, to determine the effectiveness of subacromial corticosteroid injection. This analysis is known as a factorial analysis [29] and is conducted “at-the-margins” of the table (see Table 1). The sample size for this type of analysis was calculated under the assumption that there will be no intervention interaction effect (i.e. the progressive exercise will not interact with the corticosteroid injection such that it would only work, work better or work worse when used together rather than used alone). If a substantial interaction between the two interventions is present, the factorial analysis of the groups would lead to biased results, and therefore, the efficacy of each intervention would need to be drawn from comparisons within the intervention groups (i.e. “inside-the-table” comparisons). Regardless of being able to detect a significant interaction effect, the results of the trial for the primary outcome, SPADI mean estimates, will be presented both “inside-the-table” and “at-the-margins” together with the size of the interaction [29]. The success or otherwise of the interventions will be evaluated from the analysis results conducted based on evidence for presence/ absence of a treatment interaction.

#### Interaction

An interaction between the two main treatment comparisons is not expected, but the trial is powered (at 80% and 5% (2-sided) significance) to detect a moderate standardised interaction effect size of 0.35. The presence and magnitude of an interaction (synergistic/antagonistic intervention effect [30]) between the two interventions will be formally investigated before testing their effects on the primary outcome.

An initial regression model will be fitted for the primary outcome to predict the outcome of interest and will include the two effects of interest: individually tailored progressive exercise programme versus best practice advice, subacromial corticosteroid injection versus no injection and their interaction. An estimate of the size of the interaction associated *p* value and its 95% CI will be presented.

If there is no evidence of a statistically significant interaction term (i.e. *p* ≥ 0.05), a factorial analysis will be used to determine the success of the trial. The effects for individually tailored progressive exercise programme and corticosteroid injection will be determined separately from this model as mean differences with associated 99% CI, as appropriate, adjusted for the covariates described in the “Analysis of primary outcome” section (age, sex, baseline SPADI, centre and physiotherapist effects). This model will not include an intervention interaction term.

If the interaction term is statistically significant (i.e. *p* < 0.05), the effect of individually tailored progressive exercise programme and corticosteroid injection will be evaluated from comparisons within the intervention groups: (1) ProgEx vs. BPA to test the effect of the individually tailored progressive exercise programme and (2) BPA vs. (BPA+I) to test for the effect of the corticosteroid injection. The main effects and their interaction terms will be included in the analysis model and their regression coefficient with corresponding 95% CI for the interaction terms will also be presented. The reduced statistical power of this model will be noted.

#### Analysis of primary outcome

The primary analysis will be conducted as ITT and an intervention effect group comparison on SPADI over 12 months will be evaluated. The difference in SPADI between the two intervention groups will be estimated using a repeated measures linear mixed effects regression model. The model will be adjusted for the fixed effects age and sex, baseline SPADI, and will include random intercepts by centre and observations within participant. Robust standard errors for treatment effects from all assessment time points will be reported. Clustering by physiotherapist in the progressive exercise group will be accounted for using cluster-robust standard error as part of the mixed effects model, if feasible. The unadjusted and adjusted difference in SPADI between the two types of treatment groups as well as the unadjusted and adjusted means and standard deviation will be estimated” at the margins” and “inside-the-table”. The final trial results interpretation will be based on the adjusted model. If approximate normality for the model residual terms cannot be established, a non-parametric statistical test (e.g. Mann-Whitney test for comparison of means) will be used with no adjustment, and medians and inter-quartile ranges reported instead. If parametric methods are used statistical significance will be set at the 1% level and corresponding 99% CI will be reported for the primary outcome. Floor effects in the SPADI outcome over the 12 months will be explored and reported for each treatment group. The primary outcome analysis will be conducted using the available data following item-level imputation for SPADI. Missing outcome data will be accounted for in the likelihood-based estimation of the mixed effects model of the primary analysis assuming the MAR assumption holds [28].

#### Supporting analysis based on compliance

A supplementary CACE analysis will be used to investigate the role of compliance (described in the “Description of compliance with intervention” section) in the trial effects on the primary outcome. If no evidence of a statistically significant interaction between the two treatments is identified, this analysis will be conducted at the margins using a similar mixed effects model and will be based on the CACE assumptions [31, 32]. If a statistically significant treatment interaction for the primary outcome is identified, the CACE analysis will be conducted “inside-the table”.

#### Analysis of secondary outcomes

The intervention effects on secondary outcomes will be analysed following the analysis method described for the primary outcome, on the basis that the outcomes are clinically similar. If there is no evidence of a statistically significant interaction effect for the primary outcome, then no interaction effect will be assumed for the secondary outcomes. Likewise, if evidence of a statistically significant interaction effect is identified for the primary outcome, then the secondary outcomes will be analysed assuming an interaction effect is present.

Continuous secondary outcomes analysis will be conducted following similar methods to the outline for the primary outcome analysis using linear regression for continuous outcomes and logistic/multinomial logistic regression (logit, mlogit or ologit as appropriate), for binary and ordinal outcomes. Statistical significance for secondary outcomes will be set at 5% and 95% CIs will be reported.

#### Safety

Serious adverse events (SAEs) are unlikely. All SAEs will be recorded on the GRASP SAE form and reported to the Trial Steering Committee and DMEC. SAE data will be presented descriptively as numbers and percentages for each intervention group and no statistical tests will be presented unless requested by the DMEC.

#### Pre-specified subgroup analysis

Treatment effects for the pre-specified subgroups will be analysed by utilising subgroup-by-treatment interactions for each subgroup separately and will be displayed using forest plots [33]. The following pre-specified subgroups will be analysed in line with the primary outcome analysis approach:

- Age: ≤ 64 years/≥ 65 years (rationale: increasing age has been shown to be associated with poorer outcome [34, 35])
- Sex: Male/female (rationale: prevalence is higher in males than females [1])
- Smoking status: Never smoked/former smoker or current smoker (rationale: smoking has been shown to be associated with a negative effect on tendon healing [36])
- Higher SPADI score at baseline (rationale: higher pain and functional disability at baseline may be associated with poorer outcome—we will define a higher SPADI score as ≥ 50 at baseline when SPADI converted to the 0 to 100 scale [34, 37])
- Higher Pain Self-Efficacy (PSEQ) score at baseline (rationale: higher belief in one’s ability to carry out activities despite pain may be associated with better outcome—we will define a higher PSEQ as ≥ 8 at baseline when PSEQ is converted to the 0 to 12 [38] scale).

#### Sensitivity analyses

Sensitivity analyses will be conducted to assess the robustness of the primary trial results for the ITT population in light of the assumptions made about the underlying missing data mechanism if more than 10% of data are missing. Most analyses assume data to be MAR or missing completely at random [25, 28]. The sensitivity analysis will therefore assume missing not at random, such that missing outcomes are assumed to be worse or better than the observed outcomes. The *rctmiss* command in STATA may be used for such sensitivity analysis.

### Supplementary analyses and outcomes

#### Health economics

A separate health economics analysis plan will be written by the trial health economist and all cost effectiveness analysis will be undertaken following that plan by the GRASP trial health economist.

Subsequent analyses of a more exploratory nature will not be bound by this strategy, but are expected to follow the broad principles laid down here.

### Statistical packages

All analysis will be carried out using appropriate validated statistical software such as STATA, SAS, SPLUS or R. The relevant package and version number will be recorded in the statistical report.

## Discussion

This update to the protocol contains the pre-specified SAP manuscript for the GRASP trial written according to the published guidelines on the content of statistical analysis plans [10]. The publication of the SAP aims to reduce the risk of outcome reporting bias and increase transparency of the data analysis. Any deviations or changes to the current SAP will be described and justified in the final study report and any results publications.

### Trial status

Recruitment into the trial opened on 10 March 2017 and closed on 02 May 2019. A total of 708 participants were recruited from twenty sites. Follow-up for the trial outcome data is currently ongoing and is expected to be completed by May 2020, which will then be followed by the analysis.

## Data Availability

Not applicable.

## Abbreviations

BPA: Best practice advice
CACE: Complier Average Causal Effect
CI: Confidence interval
DMEC: Data Monitoring and Ethics Committee
*EQ-5D-5L*: EuroQol 5 Dimensions 5 Levels
*EQ VAS*: EuroQol visual analogue scale
FABQ-PA: Fear Avoidance Belief Questionnaire Physical Activity
GIT: Global Impression of Treatment
GRASP: Getting it Right: Addressing Shoulder Pain
I: Injection
ICC: Interclass correlation
ISI: Insomnia Severity Index
ITT: Intention to treat
MAR: Missing at random
MCID: Minimally clinically important difference
NIHR: National Institute of Health Research
ProgEx: Progressive exercise programme
PSEQ-2: Pain Self-Efficacy Questionnaire-2
RDA: Return to desired activities
SAE: Serious adverse event
SAP: Statistical analysis plan
SD: Standard deviation
SPADI: Shoulder Pain and Disability Index
UK: United Kingdom
VAS: Visual analogue scale
+ I: Preceded by injection
